# Gas-Vapor Mixture Temperature in the Near-Surface Layer of a Rapidly-Evaporating Water Droplet

**DOI:** 10.3390/e21080803

**Published:** 2019-08-16

**Authors:** Dmitry Antonov, Roman Volkov, Pavel Strizhak

**Affiliations:** Power Engineering Institute, National Research Tomsk Polytechnic University, 634050 Tomsk, Russia

**Keywords:** high-temperature gases, water droplet, temperature field, vapor buffer layer, planar laser-induced fluorescence, laser-induced phosphorescence

## Abstract

Mathematical modeling of the heat and mass transfer processes in the evaporating droplet–high-temperature gas medium system is difficult due to the need to describe the dynamics of the formation of the quasi-steady temperature field of evaporating droplets, as well as of a gas-vapor buffer layer around them and in their trace during evaporation in high-temperature gas flows. We used planar laser-induced fluorescence (PLIF) and laser-induced phosphorescence (LIP). The experiments were conducted with water droplets (initial radius 1–2 mm) heated in a hot air flow (temperature 20–500 °C, velocity 0.5–6 m/s). Unsteady temperature fields of water droplets and the gas-vapor mixture around them were recorded. High inhomogeneity of temperature fields under study has been validated. To determine the temperature in the so called dead zones, we solved the problem of heat transfer, in which the temperature in boundary conditions was set on the basis of experimental values.

## 1. Introduction

Today, there are quite a lot of studies looking into the processes of evaporation and combustion of liquid fuel droplets [1,2,3,4]. The main consistent patterns of heat and mass transfer processes, when single droplets of liquid fuels and their aerosol flows of different particle sizes are heated and evaporate, were established by the results of theoretical and experimental research, generalized in study [5]. The influence of the temperature difference along the spherical fuel droplet radius on the intensity of heat exchange with the external gas medium was studied in papers [6,7,8,9]. Quite simple approaches have been developed to calculate the characteristics of heat and mass transfer in the “fuel droplet–gases” system [3,10]. The influence of the convective and radiative components of the heat flux on the intensity of heat and mass transfer when heating a fuel droplet in the gas medium was evaluated in study [11].

Great emphasis is given to the evaporation of large groups of water droplets and water-based compositions (solutions, emulsions, and slurries). The corresponding processes are analyzed in papers [12,13,14]. The high interest is explained by a wide use of atomized liquid compositions based on water (solutions, emulsions, and slurries) in different industrial process cycles. For instance, the research findings [13,14,15,16,17] expound the main patterns of phase transformations of water droplet flows under moderate (less than 500 °C) temperatures. However, the application fields of the evaporation processes of a large group of liquid, emulsion, and slurry droplets in gas media cover much higher temperatures (e.g., in extinguishing fires with water fog, water curtain, and water mist systems) [17].

The widely used models of liquid droplet evaporation are described in papers [13,18]. They postulate the consumption of all the energy supplied to the media interface solely on evaporation [13] (i.e., they do not take into consideration the heat sink from the droplet surface to deeper layers). These models cover quite a limited group of heat and mass transfer processes between liquid droplets and a high-temperature gas medium. As a consequence, it often becomes difficult to get a satisfactory correlation of mathematical modeling results with experimental data (especially at high temperatures of gases—over 500 °C). There are known results [17] for the comparison of integral characteristics of water droplet evaporation (evaporation rates and time of complete evaporation) in gas media of different temperatures obtained using these two models of vaporization, as well as experimental data [19]. It was established in study [17] that the most common models of water droplet evaporation are in acceptable agreement with the experimental data in the gas medium temperature range from 300 to 500 °C.

The processes of droplet entrainment by heated gases, which are difficult to control or predict [17], are one of the main problems in the generation of high-temperature (over 500 °C) gas–steam–droplet flows based on flue gases, vapors, and water droplets (e.g., in heat supply systems), as well as other liquids, emulsions, and slurries (e.g., in firefighting systems or syngas production by heating coal–water slurries). It has been a relevant task for many years to develop a predictive mathematical model to determine threshold conditions and characteristics of such entrainment in a large group of applications (in particular, in fighting fires by using fine water fog or corresponding water vapor-droplet curtains, which significantly save the consumption of fire-extinguishing agents). The high importance of solving this task is conditioned by the fact that the characteristics of droplet entrainment and evaporation in the gas medium differ greatly at various conditions of heat exchange and concentrations [17]. The most typical process in firefighting is blending of the reversed aerosol (usually water) and gases (high-temperature combustion products) flows. The task of predicting the blending characteristics (in particular, the entrainment of droplets of different sizes by gases) is complicated under phase transformations. The latter occur most rapidly at high (over 500 °C) temperatures of gases and small sizes of droplets [17]. However, there are not many experimental research findings of the corresponding processes under such temperatures. Therefore, there is a limited group of heat and mass transfer models to study these processes and their threshold conditions. Vysokomornaya O.V. et al. [17] generalized the current knowledge of phase transformations of liquid droplets in high-temperature (over 500 °C) gas media. They showed that this knowledge was gained from the classical ideas of Ranz W.E. and Marshall W.R. [20], Yuen M.C., Chen L.W. and Renksizbulut M. [15,16].

The experimental and theoretical research of the last 20 years (whose main results are generalized in the review papers [5,17]) has shown that the classical models have limitations of temperature ranges (usually, under 500 °C), for which the characteristics of evaporation processes (i.e., vaporization rates) complying with the corresponding experimental data (with 10%–15% deviations) can be obtained. For gas temperatures over 700 °C, there are no adequate models of phase transformations, making it possible to predict their rates with less than 15% deviations from the experimental data. The main reason for that is lack of corresponding reliable experimental data.

Kuznetsov G.V. et al. [21,22,23] showed that the evaporation rates of water droplets are significantly influenced by a buffer (vapor) layer around them, as well as by the unsteady and inhomogeneous nature of the temperature field generation of evaporating droplets. Based on the experimental data from [23,24], hypotheses were formulated about the unsteadiness and inhomogeneity of temperature fields of evaporating droplets, the dominance of their thermal insulation effects (limitations of the values of thermal flows supplied due to rapid vaporization). It is a pressing task to provide experimental grounds for the formulated hypotheses [23,24]. This will make it possible to develop models to predict the evaporation rates of liquid droplets in a wide temperature range (up to 2000 °C), covering all the promising heat technologies. It makes sense to define the characteristics (thickness of layers, temperature gradients, gas composition, etc.) of thermal (vapor) insulation of liquid droplets, solutions, and slurries when they move in high-temperature gas media.

The fundamental findings of such studies will make it possible to significantly supplement the present-day ideas of high-temperature evaporation of liquid droplets. As a result, recommendations will be developed to optimize the operating procedures in which these effects are involved. The potential optimization implies minimizing the energy and time when realizing phase transformations and increasing the fullness of evaporation of liquids. In addition to thermocouple measurements [23,24] it is reasonable to use optical techniques of high-speed tracer recording planar laser-induced fluorescence (PLIF) [25,26,27,28,29] and laser-induced phosphorescence(LIP) [30]. Such approach will make it possible to obtain dozen of times more temperature values in the points of medium around an evaporating droplet and in its near-surface layer. High-speed recording of such values will enable to frame the concept of changing temperature and concentration (of water vapor) fields around evaporating water droplets. The main problem with PLIF and LIP is that they do not provide the recording of temperatures in a thin vapor layer near the medium interface (i.e., the surface of a rapidly-evaporating liquid droplet). Therefore, the only way to plot a temperature field in the region inaccessible for measurement is to solve the problem of heat transfer with boundary conditions, using the results of temperature measurement in the points at the interfaces of this region, which are accessible for PLIF and LIP techniques.

The purpose of this research is to record unsteady temperature fields in the near-surface layers of a rapidly-evaporating water droplet and in a thin region around it, using LIP and PLIF to expand the concepts of a vapor buffer coating formation around the droplet.

## 2. Experimental Setup and Procedure

**Figure 1:**
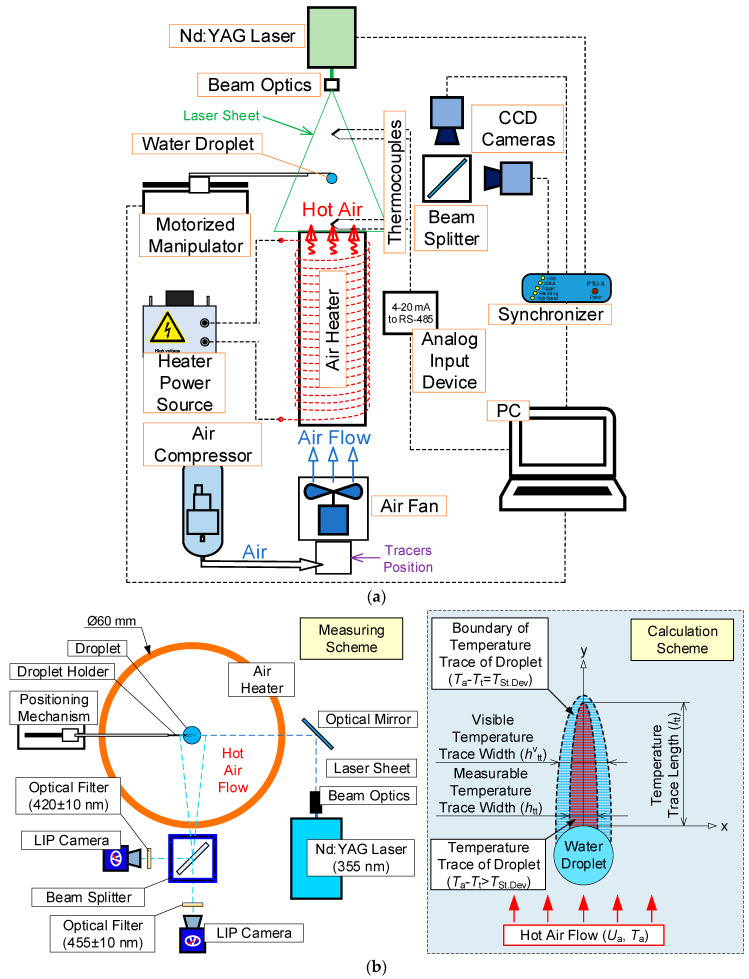

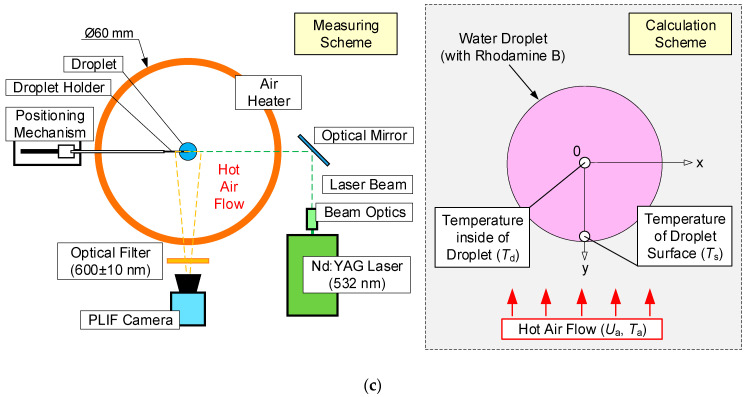
(**a**) Scheme of experimental setup, (**b**) laser-induced phosphorescence (LIP) measurement, (**c**) planar laser-induced fluorescence (PLIF) measurement.

Unlike in the experiments [23,24], in this study we focus on non-contact measurement of temperature and velocity of a gas-vapor mixture in the near-surface vapor layer of a water droplet, as well as the temperature of the latter. Since the optical measurement techniques PLIF and LIP have some limitations of the observation area sizes (due to the glare effect, errors of calibration curve plotting, threshold values of temperature measurement considering the properties of the fluorophores used, and solid particles) near the media interface, it is reasonable to make additional calculations using the model of high-temperature water droplet evaporation [22] and taking into account the dependence of vaporization rate on temperature, obtained in study [21]. We used a setup schematically represented in Figure 1.

### 2.1. Heated Air Flow Parameters

Similarly to the experiments in studies [23,24], we used a compressor and specialized thermal elements to provide the conditions of heating a water droplet in a hot air flow or in an almost motionless air medium with a temperature from 20 to 500 °C. To generate a hot air flow, we used a system (Figure 1) featuring an axial air fan with an in-built rpm control and an insulated ceramic tubular heater. The latter was a vertical heat-resistant ceramic cylinder (1 m high, 60 mm in outer diameter, and 52 mm in inner diameter) with energized nichrome wires wound on it. The air flow had the following parameters in the experiments: *U*_a_ = 0.5–6 m/s and *T*_a_ = 20–500 °C.

### 2.2. Water Droplet

In the experiments, we used droplets with a radius *R*_d_ of 1–2 mm and a volume *V*_d_ ranging from 5 to 30 μL. Droplets were generated using a single-channel Finnpipette Novus dispenser with a volume increment of 0.01 µL. The resulting droplet was placed on a hollow steel holder tube, 0.3 mm in outer diameter and 0.2 mm in inner diameter. The holder was fixed on the positioning mechanism with a copper tube. The motorized mechanism was a horizontal mobile construction controlled by a PC operator. A droplet fixed on a holder was introduced into the hot air flow at about 0.15 m/s. The positioning accuracy was 0.05 mm. The droplet was stopped when it reached the vertical symmetry axis of the ceramic heater at a distance of about 30 mm relative to its upper part (Figure 1). At that point, we started the photo and video recording of the processes under study.

### 2.3. Plotting Temperature Fields Using LIP

The temperature of the air flow before its contact with an evaporating droplet and gas-vapor mixture in the trace of the latter was determined using LIP [30]. Figure 1b (on the left) shows the scheme of the experiment. The method is based on the variation of luminosity (emitted laser light) of the phosphorus indicators exposed to heat. The air flow was seeded with BaMgAl_10_O_17_:Eu (BAM:Eu) particles, 6–8 μm in size. The upper threshold temperature, at which BAM:Eu particles remain stable (do not thermally decompose), does not exceed 600 °C. The emission spectra of the tracer particles used are described in [30]. When implementing the LIP technique, we used the following equipment (Figure 1b): a single-pulse Nd:YAG laser Quantel Q-smart 450 (wavelength 355 nm, pulse frequency 10 Hz, pulse energy 130 mJ), two charge coupled device (CCD) video cameras Imager M-lite 2M (frame resolution 1920 × 1280 pix, frame rate 10 fps, digit capacity 12 bit), and an optical beam splitter with a dichroic reflector. The photos were taken using two macro lenses Sigma DG 105 mm f/2.8 EX Macro (the focal ratio was set at 2.8). In accordance with the features of the technique [30], we used two color filters: 420 ± 10 nm and 455 ± 10 nm to record changes in the intensity of light emitted by BAM:Eu particles corresponding to the right (~455 nm) and left (~420 nm) sides of the spectrum, respectively. This approach implies determining the temperature as a function of the intensity ratio of two images—420/455 nm. At the same time, the intensity of the light emitted by the particles at ~420 nm largely depends on the flow temperature (LIF-T channel). Recording the light emitted by the particles at ~420 nm makes it possible to account for the relatively inhomogeneous particle concentration in the measurement area (LIF-C channel).

**Figure 2:**
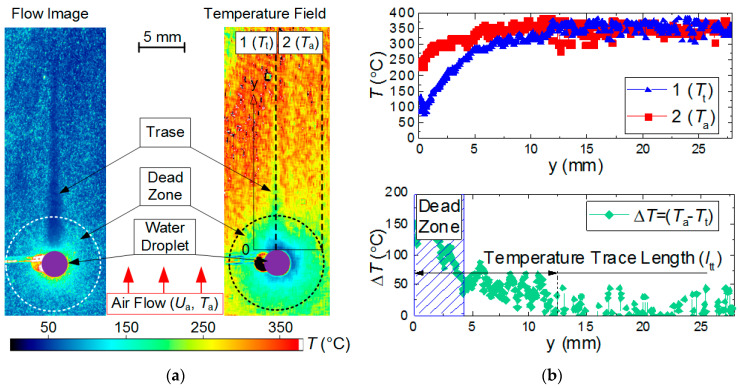
(**a**) Primary results of measurement and (**b**) processing with LIP.

Figure 2 presents typical frames, the results of measurement, and their processing using LIP. The error of the gas-vapor mixture temperature measurement using LIP depended on the temperature of the controlled gas medium, and did not exceed 15 °C for the range under study.

To correctly process the results of non-contact measurement using LIP, preliminary experiments were conducted to plot calibration curve (illustrating the dependence of the temperature of the object under study on its luminous intensity when BAM:Eu particles were added). The experiments on plotting the calibration curve were conducted in the following way. Without a water droplet in the frame, we recorded the images of the flow with the moving BAM:Eu particles. The temperature in the recording area (*T*_a_) was calculated as the arithmetic mean between the readings of two type S (platinum–platinum–rhodium) thermocouples (temperature range 0–1200 °C, accuracy ±1 °C, junction diameter 0.05 mm and thermal lag 0.1 s) installed at the input and output of the recording area (Figure 1a). The difference between the thermocouple readings ranged from 5 to 16 °C depending on the flow temperature. This is why the maximum error of the temperature *T*_a_ in the experiments was ±8 °C. For each *T*_a_ value, we determined the resulting ratio of the recorded emission intensities of the BAM:Eu particles—420/455 nm—and plotted the calibration curve. Then, with the similar experiment settings, experiments were conducted to record the temperature field in the trace of an evaporating droplet using the DaVis software. The resulting ratios of the recorded emission intensities (420/455 nm) were recalculated into the temperature (*T*_a_) using the calibration curve. The accuracy of temperature measurement using the LIP technique ranged from 4% to 7% [30].

### 2.4. Plotting Temperature Fields Using PLIF

The PLIF technique was used to record the temperature distributions within an evaporating water droplet. The scheme of the experimental setup is shown in Figure 1c. Droplets were made from the water solution of the Rhodamine B fluorophore (as in [31]) in a mass concentration of 1000 μg/L. This fluorophore has a pronounced dependence of the emission intensity on the liquid temperature (it decreases by 2% per 1 °C) under 532 nm light irradiation.

When implementing the PLIF technique, we used the following equipment (Figure 1c): a double-pulse Nd:YAG laser Quantel EverGreen 70 (wavelength 532 nm, pulse frequency 4 Hz, pulse energy 74 mJ) and a CCD video camera ImperX IGV-B2020M (frame resolution 2048 × 2048 pix, frame rate 4 fps, digit capacity 16 bit). A droplet was scanned along its vertical symmetry axis by a light sheet. The thickness of the light sheet did not exceed 0.2 mm in the measurement area. The droplet was photographed using a macro lens Nikon Micro-Nikkor 200mm f/4D ED-IF A with a pre-installed color filter of 600 ± 10 nm. The color filter made it possible to record the light irradiated by the Rhodamine B dye near the maximum emission spectrum and to eliminate the unwanted 532 nm light reflected from the droplet.

As with the LIP technique (Section 2.3), the experiments had two stages. The first one consisted of plotting a calibration curve; the second one involved the recording of the temperature fields of the evaporating droplets. We used the Actual Flow software for it. When plotting a calibration curve, we determined the correlation between the intensity of the light emitted by the dye and the current water temperature (*T*_d_). A water droplet was introduced into the air flow and the air temperature varied in the following range: *T*_a_ = 20–500 °C. With each fixed *T*_a_ value, the temperature in the droplet center gradually approached the steady-state value [31]. To determine the current value of the temperature *T*_d_ at the calibration stage, we introduced a fast type S thermocouple (0.05 mm in junction diameter, with a 0.1 s time lag, and with ± 1 °C) into the droplet. A series was recorded consisting of 30 to 150 images for each *T*_d_ value depending on the water evaporation rate. In each frame obtained, we averaged the luminous intensity of Rhodamine B in an area of no more than 0.5 × 0.5 mm around the thermocouple junction in the droplet. After that, we found the arithmetic mean of the luminous intensity of Rhodamine B around the thermocouple junction in all the frames for a certain *T*_d_ value. Then we plotted the resulting temperature calibration curve. With the similar equipment settings, we conducted experiments to record the temperature field in the section of an evaporating water droplet. The resulting droplet images were recalculated into the temperature values (*T*_d_) using the calibration curve plotted earlier.

**Figure 3:**
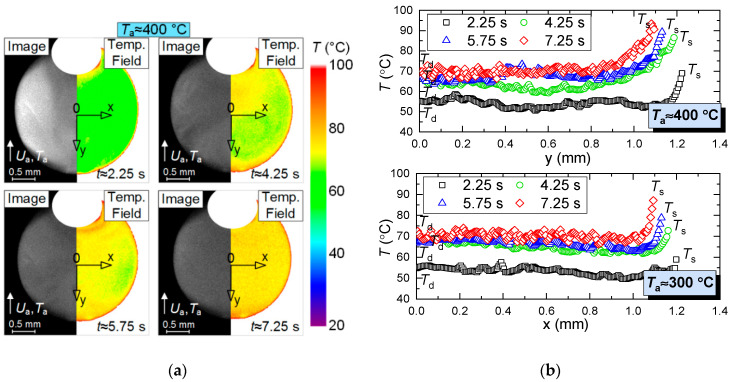
(**a**) Primary results of measurement and (**b**) processing with PLIF.

The systematic errors of determining the temperature field of an evaporating water droplet using PLIF did not exceed 2 °C. Strizhak P.A. et al. [31] present the results of a detailed study into the temperature fields of evaporating droplets when varying the main parameters (in particular, initial temperature and velocity of the incoming air flow, as well as droplet dimensions). The limitations of using PLIF technique were defined [31] to meet the set objectives (including the ones in this research). Primary results of PLIF measurement are shown in Figure 3.

### 2.5. Plotting Velocity Fields of Gas-Vapor Mixture Using PIV

Particle image velocimetry (PIV) was used to determine the velocities of the heated air flow (*U*_a_) and gas-vapor mixture in the droplet trace (*U*_t_) as well as to study the parameters of the latter. The laser and video camera used here were similar to those used for the PLIF technique (Section 2.4). Unlike for the PLIF technique, here we used the Sigma DG 105 mm f/2.8 EX Macro lens. The air flow was seeded with particles of TiO_2_ powder, 0.1–2 μm in size. We recorded the images of tracing TiO_2_ particles around the evaporating water droplets. The size of the recording area was 60 × 60 mm. The recording frequency was 10 fps, with a pair of frames recorded for each moment of time (the maximum delay between two frames was 70 μs in all the experiments). For each initial value of *R*_d_, *U*_a_, and *T*_a_, an experiment consisted of 200–1000 pairs of frames, depending on the droplet lifetime in the flow. At least five experiments were conducted for identical values of *R*_d_, *U*_a_, and *T*_a_. The resulting frames were analyzed using a cross-correlation image processing algorithm. At this stage, each image of the pair was divided into connected elementary domains 32 × 32 pix each. Within each domain, we searched for the most probable shift of the tracing particles during the time of the delay between two frames. For each domain, we plotted the velocity vector of the tracing particles. Thus, we produced a regular two-component instantaneous flow velocity field for each frame pair, consisting of 4096 vectors at grid points. To smooth the resulting fields, we averaged each 10 consecutive instantaneous velocity fields: An average velocity vector was found for each separate elementary domain. Ultimately, the averaged two-component velocity fields obtained with a 1 s increment were included in the post-experiment analysis. The error of *U*_a_ and *U*_t_ velocity estimation did not exceed 2%.

### 2.6. Calculated Parameters of Processes under Study

When processing the experimental results, we determined the values of variations of temperature (Δ*T* = *T*_a_ − *T*_t_) and velocity (Δ*U* = *U*_a_ − *U*_t_) of the air flow in the near-surface layer of an evaporating water droplet. We determined the main characteristics of the thermal trace: length *l*_tt_, width *h*_tt_ at different distances from the droplet (5–25 mm), gas-vapor mixture temperature in the trace *T*_t_. The dimensions of the temperature trace were measured in two stages. At the first stage, we determined the temperature trace boundary and at the second one, its dimensions. The temperature trace of a droplet was the area where the inequality *T*_a_ < *T*_t_ held true. The temperature trace boundary was defined as the area in which the equation *T*_a_ − *T*_t_ = *T*_St.Dev_ held true, where *T*_St.Dev_ is the systematic error of the LIP technique (Figure 1b, on the right). After identifying the temperature trace boundary, we calculated the values of *l*_tt_, *h*_tt_, and Δ*T*. To estimate the values of *l*_tt_ and Δ*T,* we used an approach implying the plotting of two independent vertical sections (Figure 2): on the symmetry axis of the water droplet (obtaining *T*_t_); at 5–10 mm from the symmetry axis (obtaining *T*_a_). For each point of y axis, the temperature difference was calculated: Δ*T* = *T*_a_ − *T*_t_. The method to determine the thermal trace width *h*_tt_ is similar to the one described above. The same approach was used to determine the characteristics of the aerodynamic trace of a droplet. The domain, where the *U*_t_ values did not differ from the *U*_a_ values by more than 2% (systematic error of the PIV technique) was taken as the aerodynamic trace boundary.

## 3. Results and discussion

### 3.1. Unsteady Temperature Fields of an Evaporating Droplet and Gas-Vapor Mixture around It

**Figure 4:**
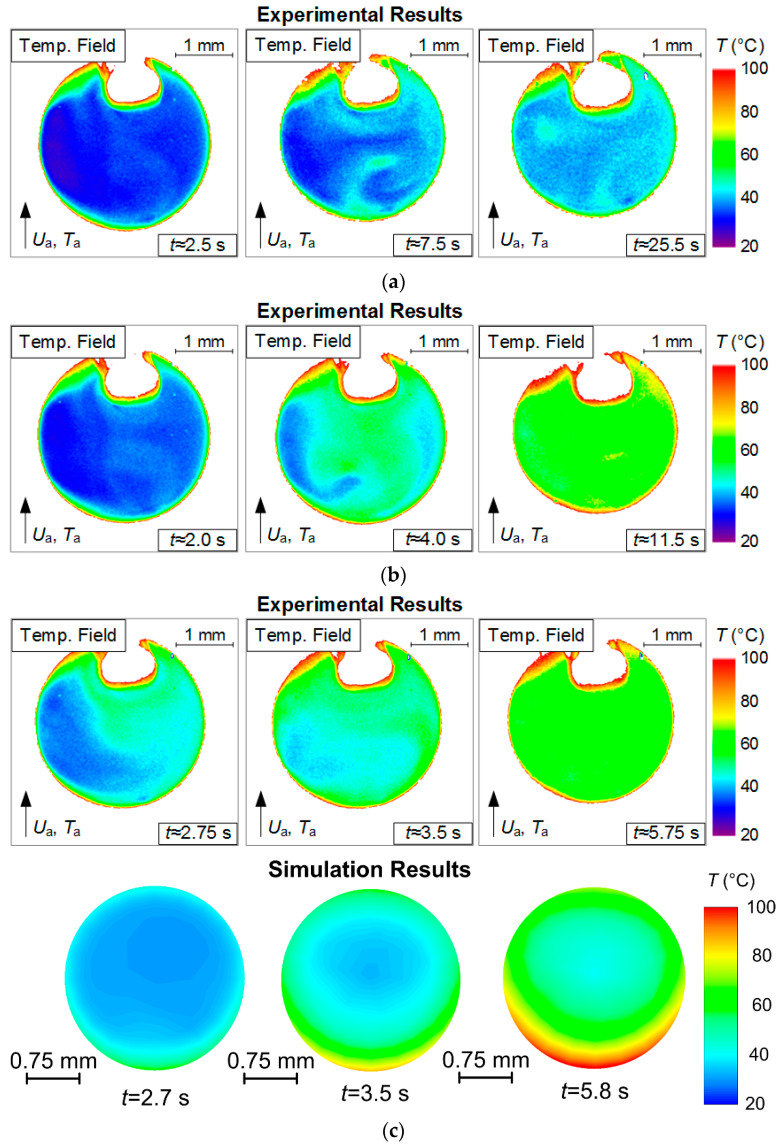
Experimentally-obtained unsteady temperature fields of an evaporating droplet and around it at different temperatures of gas flow (*U*_a_ ≈ 5 m/s; *R*_d_ ≈ 1.53 mm): (**a**) −100 °C, (**b**) −200 °C. The results of modeling with the algorithm from [22] at *T*_a_ ≈ 200 °C (**c**).

**Figure 5:**
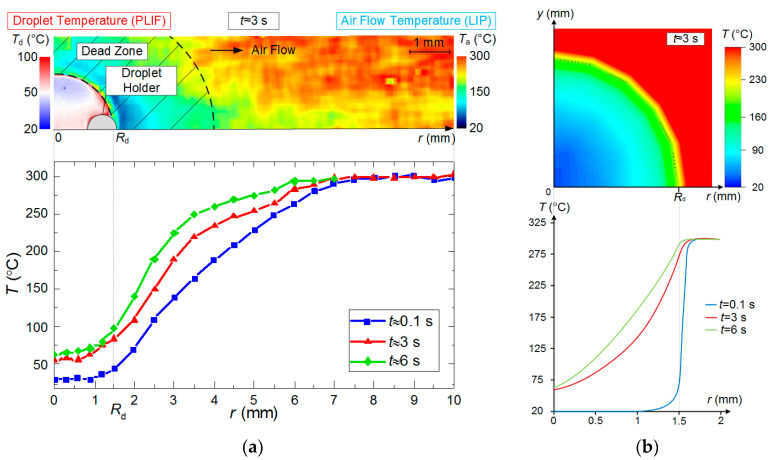
Temperature distribution along longitudinal sections of the observation area at different points of time; the results of processing the experimental data with PLIF and LIP are presented. (**a**)—experiment, (**b**)—modeling (*T*_a_ ≈ 300 °C; *U*_a_ ≈ 5 m/s; *R*_d_ ≈ 1.53 mm).

**Figure 6:**
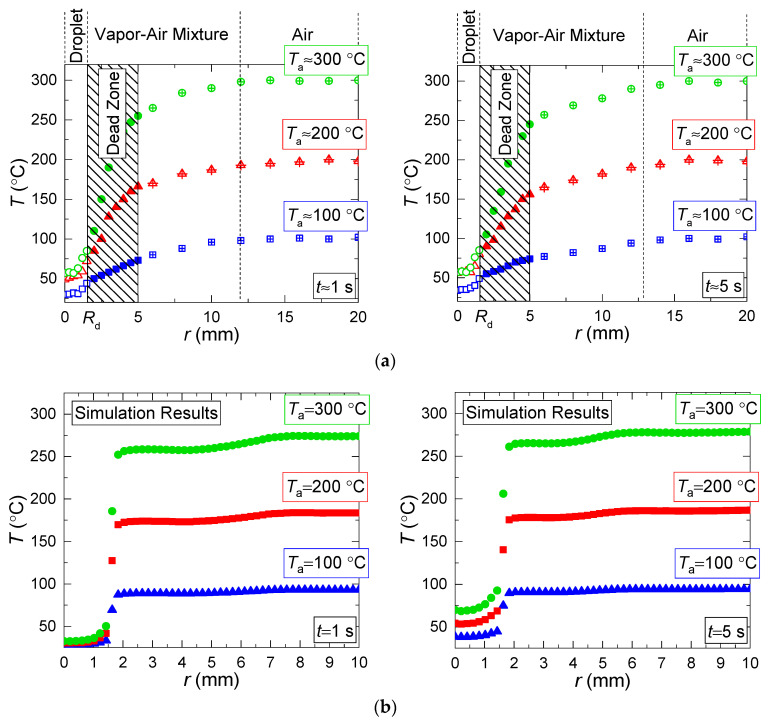
Temperature distribution along longitudinal sections of observation area at different temperatures of heating and at different points of time: (**a**)—experiment, (**b**)—modeling (*U*_a_ ≈ 5 m/s; *R*_d_ ≈ 1.53 mm).

**Figure 7:**
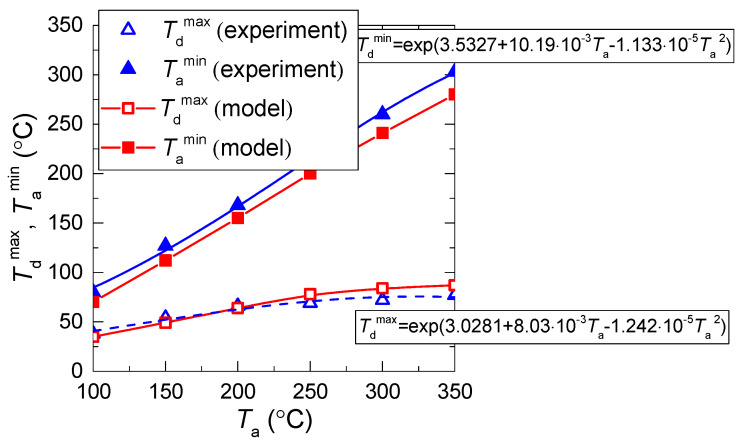
Threshold (minimum and maximum) temperature values reached by evaporating water droplet and in its trace (*U*_a_ ≈ 5 m/s; *R*_d_ ≈ 1.53 mm).

Figure 4, Figure 5, Figure 6 and Figure 7 present typical temperature fields of evaporating water droplets, as well as of a gas-vapor mixture around them and in their trace. The latter is most distinct when the velocity of the incoming hot air flow increases. When analyzing Figure 4, Figure 5, Figure 6 and Figure 7, we determined the corresponding changes in the temperature and velocity of the gas-vapor mixture in the near-surface layer of the droplet relative to the incoming flow. An overall good correlation could be noted between the primary experimental results (Figure 4) and the data from [31]. The main difference is in the droplet surface profile, as in the experiments in [31], the recording was made in the section perpendicular to the one selected in the present study. Though if we analyze the absolute values of water droplet temperature, their difference from those in study [31] under identical heating conditions does not exceed 5 °C. Therefore, we decided to further focus on a complex analysis of the temperature of the vapor layer forming between the heated air and the droplet without a detailed consideration of the aspects of droplet heating and its transition from a highly inhomogeneous to a quasi-steady temperature field. These aspects are considered in study [31]. Using droplets of different initial sizes, Strizhak P.A. et al. [31] show that depending on the conditions of energy supply, the times of droplet heating can reach 10% to 15% of the total evaporation time of droplets. Thus, a droplet can have a quasi-steady temperature field for most of the evaporation time [31]. In this study, we used a simplified statement of the problem. A full mathematical model accounting for the impact of the holder on the heating and evaporation of the droplet (controlling for the contact area and droplet shape) was presented in our earlier paper [31]. There we showed that accounting for a holder (as exemplified by steel rods, 0.3–0.5 mm in diameter) will make it possible to correct the heating and evaporation times of water droplets by no more than 5% to 8%. Here, we used a hollow steel tube, 0.3 mm in the outer diameter and 0.2 mm in the inner diameter, as the droplet holder. Therefore, this factor is negligible in the present study.

The most significant temperature values are those that a droplet is heated to before rapid evaporation, because these values of the temperature field determine the vaporization rates and, consequently, the concentration of vapor in the near-surface (wall) layer of the droplet. The evaporation of a droplet with a quasi-steady temperature field for a long time is explained by the common mechanism of its heating under phase transformations. In particular, a droplet has relatively low (20–30 °C) temperatures early in the process. The vaporization rates at such temperatures of the droplet surface are extremely low. Consequently, the energy supplied to the droplet surface is almost completely spent on heating the droplet, with only a small part of it spent on the endothermic phase transition. As the droplet heats up, the temperature increases and so do the vaporization rates. This leads to the redistribution of energy and increase in the share of energy spent on phase transformations. Since the heat of water evaporation is extremely low (2.26 MJ/kg), little energy remains to heat the droplet. This results in slower heating of the droplet, and it continues to evaporate with a quasi-steady temperature field. Only small fluctuations with a few degrees were recorded, which remained within the measurement error.

Based on the experimental results in Figure 5, Figure 6 and Figure 7, we can make several conclusions:

- First, the limitations of LIP in the recording of the temperature of the gas-vapor mixture around the droplet (Figure 5) have been established and viability of using the model from [22] is shown to correct the gas-vapor mixture temperature around the water droplet surface (i.e., in the dead zones of LIP). In general, applying interpolation and extrapolation methods, we can use the experimental points obtained with LIP in the gas-vapor area and with PLIF in the droplet to predict the temperatures in the gas-vapor layer around the droplet. Figure 5a,b shows that the temperature profiles in the experiments and when modeling are also in good agreement for the areas where it is impossible to plot reliable experimental fields; the prediction can be made using temperature distribution obtained by means of the model. These procedures were used when plotting such temperature distributions in Figure 6 with experimental values *T*_d_ and *T*_g_. Figure 7 shows that the absolute maximum temperature values in reference points coincide.
- Second, we clearly show the unsteady changes of temperature fields of heated and rapidly-evaporating water droplets (Figure 5 and Figure 7). A similar conclusion was made from the experimental results in study [31]. This factor is extremely important and determines the conditions and characteristics of water droplet heating when energy is supplied and spent on the phase transition. Traditionally, assumptions are made [12,13,14,15,16] that all the energy supplied to the water droplet surface is spent on the phase transition, and the process of its heating is not decisive. However, the temperature fields obtained illustrate that a water droplet has a highly inhomogeneous temperature field for a long time (temperature variations can be 70 to 80 °C). Therefore, it is important to take this factor into consideration in the mathematical modeling. The temperature fields, obtained when combining the results of processing using LIP and PLIF, show that the supplied energy is spent not only on the phase transition but also on heating the water droplet and the gas-vapor mixture. The heating of the gas-vapor mixture is a complex process: on the one hand, the energy of the air flow is spent on the heating of relatively cold vapors injected from the droplet surface, and on the other, the water vapors are known to contribute to additional radiation. Hence, in actual operating conditions, the vapors forming in the near-surface layer of an evaporating droplet strengthen the radiative heat exchange.
- Third, combining the temperature fields of PLIF and LIP brings about difficulties in the adequate control of temperature in the thin near-surface layer (Figure 5), as we plotted the temperature fields of the gas-vapor mixture and droplet within several pixels for the media interface. Therefore, it was reasonable (Figure 5 and Figure 6) to use an interpolation approach in this small-size region (i.e., plot temperature and temperature distribution fields for the dead zones of PLIF and LIP using the values in the extreme points of reliable regions in the liquid and vapor phases).

**Figure 8:**
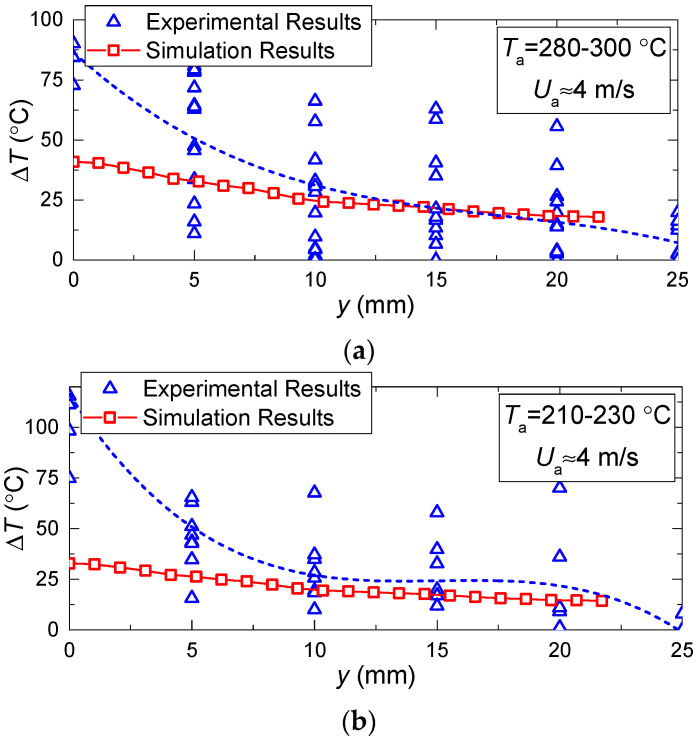

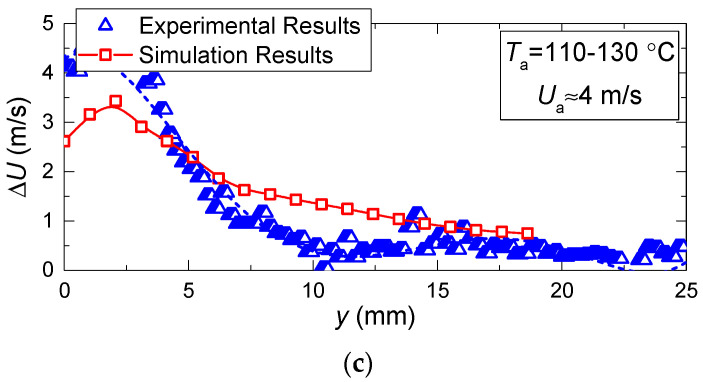
Temperature and velocity variation in the trace of evaporating water droplet vs. relative distance from the droplet (*V*_d_ ≈ 15 μL, *R*_d_ ≈ 1.53 mm) at constant *U*_a_ ≈ 4 m/s and different *T*_a_: (**a**)— *T*_a_ ≈ 280–300 °C, (**b**)— *T*_a_ ≈ 210–230 °C, (**c**)— *T*_a_ ≈ 110–130 °C.

**Figure 9:**
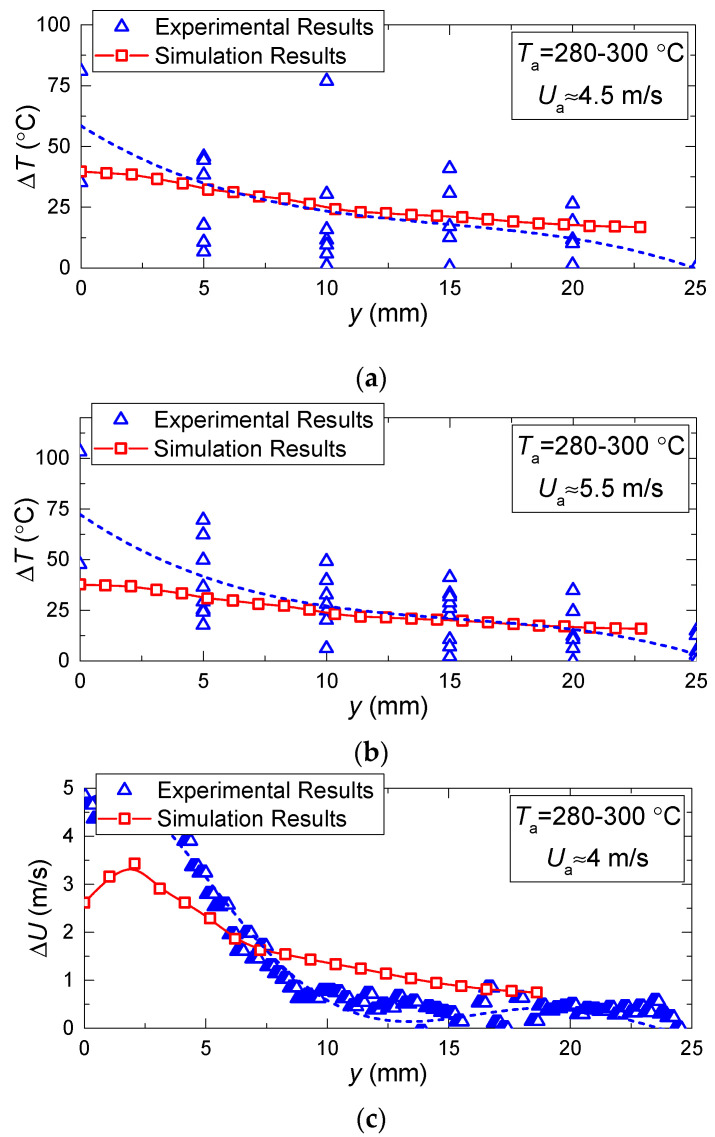
Temperature and velocity variation in the trace of evaporating water droplet vs. relative distance from the droplet (*V*_d_ ≈ 15 μL, *R*_d_ ≈ 1.53 mm) at limited change of *T*_a_ ≈ 280–300 °C and different *U*_a_: (**a**)— *U*_a_ ≈ 4.5 °C, (**b**)— *U*_a_ ≈ 5.5 °C, (**c**)— *U*_a_ ≈ 4 °C.

Figure 8 and Figure 9 present the values of temperature and velocity variations as functions of time, established experimentally and through mathematical modeling. It is clear that the dispersion of experimental points is quite moderate (does not exceed 15%–25% relative to the value corresponding to the approximation curve). This result is conditioned by a satisfactory repeatability of heating conditions and measurements in the conducted experiments. In general, there is good agreement between the experimental and theoretical values of the temperature and velocity variations of the gas-vapor mixture under study. A good correlation for the velocities of the gas-vapor mixture, established when modeling and experimentally, makes it possible to conclude that the model [22] very well describes the convective heat exchange near the media interface. Deviations in the theoretical and experimental values of the gas-vapor mixture temperature at a small distance from the droplet surface show that the model [22] does not make it possible to describe the real-life conditions of rapid vapor jetting from the droplet surface. The temperature variation in the trace of an evaporating water droplet in the experiments was much higher than when modeling.

Figure 8 and Figure 9 present the typical areas of moving away from the droplet surface, for which the gas-vapor mixture temperature was reliably recorded using LIP. Dead zones emerge at the droplet surface because the phosphorus particles stick to the droplet, thus changing their concentration around it. This dead zone around a droplet was 2–4 mm wide. Due to their small size and low concentrations, the tracers reaching the droplet surface could not have a significant impact on the droplet heating and evaporation rates. Moreover, most of the tracers stuck to the droplet on the side of the incoming flow. Our experiments estimated how tracers affect water droplet evaporation rates using the video frames (we used a Photron SA1.1 video camera with a frame rate of 100 fps), while monitoring the variation rate of the droplet size due to vaporization. We conducted two series in equal conditions. In the first series, tracers were not injected into the gas flow, and in the second one, they were. We measured the times of full droplet evaporation with an accuracy of 0.01 s. The average time of complete evaporation for a droplet of clear water was found to be 35.9 s, and for a droplet with the BaMgAl_10_O_17_:Eu (BAM:Eu) particles stuck to it was 37.8 s. Thus, the average difference in the complete droplet evaporation time for these two cases was 1.9 s or 5.2%. Similar experiments involved the air flow with titanium dioxide particles moving around water droplets. The differences in the droplet evaporation properties were less than 3%.

The temperature variations in the trace of evaporating water droplets are rather high in the experiment because phosphorus particle seeding according to the LIP method can be inhomogeneous, which may lead to fluctuations in their concentration and luminosity. These distort the temperature field, especially near a rapidly-evaporating water droplet. The velocity variations in the trace of evaporating water droplets also stem from the inhomogeneous seeding of the tracer particles of TiO_2_. These factors are difficult to account for in a model, since they result from particles sticking to the droplet and their entrainment from the droplet surface with the gas flow. Therefore, the velocity and temperature fields obtained by modeling illustrate a smoother change than in the experiments.

**Figure 10:**
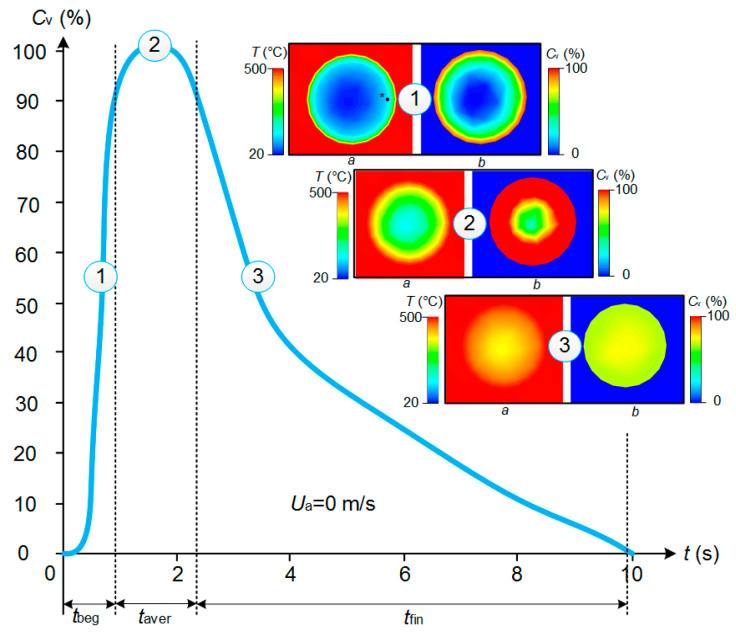
Temperature (**a**) and concentration (**b**) fields around an evaporating droplet, obtained by using the model from [22].

Figure 8 and Figure 9 show that the higher the temperature of the incoming air flow, the more distinct the deviations in the experimental and theoretical variations of temperature in the near-surface gas-vapor layer. This result again confirms a decisive effect of vaporization on the formation of a buffer layer around the droplet and growth of the width of this layer with a corresponding increase in the concentration of water vapors. This factor becomes most noticeable when the temperature increases and the velocity of the incoming hot air flow decreases. We can conclude that a complex of sign-variable processes occur in the near-surface layer of an evaporating water droplet: diffusion of vapors, their heating, intensification of radiative heat exchange due to self-radiation of water vapors, convective heating of the droplet, shift of the media interface resulting from water evaporation, transformation of the droplet surface, etc. All these effects are difficult to take into account in the mathematical modeling. Therefore, it makes sense to highlight the main factors and processes. Figure 10 presents the fields of temperature and vapor concentration in the near-surface layer of an evaporating water droplet.

Figure 10 shows that the concentration of water vapors is close to the maximum possible near the droplet surface. It is impossible to record such fields experimentally. Therefore, we had to use the model from [22] for a comparative analysis. Figure 8 and Figure 9 show that when the temperature of the oncoming air flow increases, the temperatures of the gas-vapor layer around the droplet become significantly higher in the experiments, than when modeling. Thus, the results of mathematical modeling shown in Figure 10 can be considered the lower estimates of the real ranges of temperature changes around the media interface under study. The higher the heating gas medium temperature, the more distinct the effect of the thermal (vapor) insulation in the near-surface droplet layer.

### 3.2. Generalized Curves

**Figure 11:**
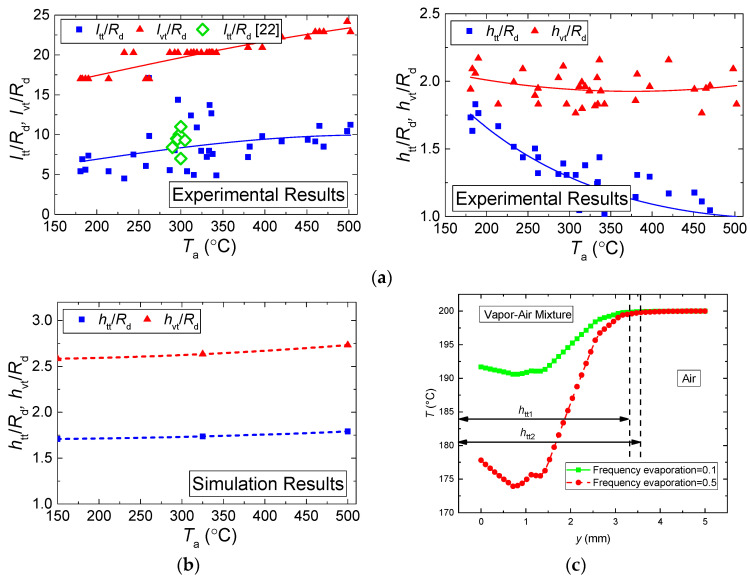
Relative ratios of the length of the droplet trace (aerodynamic and temperature) to droplet size (**a**) (comparison with the experimental data [24]) and to the thickness of the vapor buffer layer around the droplet (**b**), calculated by the modeling results at *U*_a_ ≈ 5 m/s; *R*_d_ ≈ 1.53 mm; *t* ≈ 5 s (**c**) width of the temperature trace depending on the evaporation rate.

Figure 11 presents the relative sizes of vapor layers formed around an evaporating water droplet and in its trace. It also shows the values of the same parameters obtained using thermocouple measurements [14,15,16]. Overall, we can see an acceptable correlation between the experimental and theoretical values of these characteristics. However, if we compare the experimental data obtained using thermocouples and non-contact methods, the influence of the unsteadiness of droplet heating followed by changing dimensions of the evolving vapor layer around the droplet becomes most noticeable.

The dependences of the buffer layer thickness *h*_vt_ on the temperature *T*_a_, calculated by the modeling and by the experimental results, when the velocity fields are analyzed, correlate well both in the curve slopes and in the ranges of changes of the corresponding values (Figure 11). This is explained by a satisfactory correlation of velocity fields around an evaporating droplet, established when modeling and when using PIV in the experiments. The curves for the thickness of identical layers, established when analyzing the temperature fields, *h*_vt_ in Figure 11b are similar in the slope in the experiment and when modeling, though differ in the absolute values almost 1.5 times.

The modeling values of *h*_vt_ are higher than the experimental ones. This is conditioned by the fact that, when modeling, the evaporation rate of a water droplet was calculated depending on the gas-vapor media temperature, whereas in real-life conditions, its value is affected not only by the temperature, but also by the concentration of vapors around the media interface. Therefore, the evaporation rate and, consequently, the concentration of vapors when modeling were somewhat higher, and the values of *h*_vt_ exceeded the identical parameters established in the conducted experiments. The mathematical modeling shows that the higher the gas medium temperature, the more rapidly water droplets evaporate. Therefore, more water vapors emerge and the temperature trace (i.e., the area with a lower temperature of the gas-vapor mixture vs. the incoming flow temperature) becomes wider. In the experiment, the evaporation rate is affected not only by the temperature but also by the concentration of vapors around the droplet, which slows down droplet evaporation and decreases the trace size. Figure 11c shows the change of the temperature trace with varying frequency evaporation. The results are presented for the frequency evaporation = 0.1 and 0.5. Clearly, the higher the evaporation rate, the larger the width and length of the temperature trace. 

In [31,32], we provide the instantaneous and mean evaporation rates of water droplets exposed to high-temperature heating obtained experimentally and using mathematical modeling. We obtained the curves of these velocities versus the temperature of the external gas medium and the temperature of the droplet surface. To calculate the average evaporation rates, we monitored the variation rate of the droplet radius throughout its lifetime. Instantaneous evaporation rates were calculated with due consideration of the short time intervals at any stage of the droplet lifetime. The mean droplet evaporation rates at the gas medium temperature ranging from 300 to 800 °C fall into the range of 0.03–0.15 kg/(m^2^s). The mean vaporization rates were lower than the instantaneous ones, since they accounted for the whole droplet heating and evaporation time. The comparative analysis of the findings of this study with the data from [31,32] in terms of full droplet evaporation times shows that they are in quite a good agreement—the deviations do not exceed 12%. To provide such an acceptable agreement, it is necessary to set the frequency evaporation values in the model based on the experimental data on the evaporation rates from [31,32].

**Figure 12:**
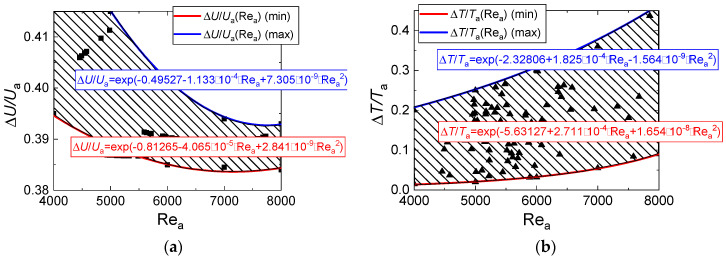
Relative variations of rate (**a**) and temperature (**b**) in the vapor buffer layer around droplet and in its trace vs. initial values at different temperatures and air flow velocities (as generalized dependences on the Reynolds number Re_a_ = 2 *U*_a_*R*_d_/ν_a_). The experimental points are market in the figures.

Figure 12 presents the relative variations of temperature and rate in the near-surface layer of evaporating water droplets with typical illustrations of approximation equations. The latter can be used to predict the influence of each of the factors, processes, and effects under study. It clearly shows the contribution of nonlinear dependences of evaporation rates on the temperature and dimensions (explored in studies [31,32]).

The research findings develop the earlier ideas on the temperature and aerodynamic traces of evaporating droplets formulated in [33] by combining mathematical modeling and experimental research. Due to such combined usage of theoretical and experimental data, we managed to predict the velocities and temperature of the gas-vapor mixture in the close vicinity of the droplet surface for the first time, where the known contact and non-contact methods cannot provide reliable experimental measurements. This is what makes the scientific novelty of this research. In the future, this study can be developed to research the heating and evaporation of a group of droplets located next to each other and determine their impact on Δ*T* and Δ*U* around each droplet and behind them as a group. Such research findings are important for studying the temperature and aerodynamic traces of aerosol flows or fine droplet clouds. The conditions and rates of liquid heating and evaporation in these cases [34] differ greatly from the similar parameters obtained in the experiments with single droplets, as shown in this research and in [31,32]. The experimental data obtained are objective premises for the development of adequate mathematical models of heat and mass transfer, enabling us to predict the necessary and sufficient conditions of complete evaporation of water droplets in a flame (or flow of heated gases) with due consideration of the formation of a thermal (vapor) insulation, transformation, and slowdown of droplets moving through high-temperature combustion products or other gas media. The solution of this problem, as illustrated by single droplets, is useful to gain new fundamental knowledge of the physical aspects of water evaporation in a hot gas flow, and to understand how the contribution of a vapor cloud to fighting fires can change when the volume of injected water increases greatly. In general, the research findings are important for the development of promising high-temperature gas-steam-droplet applications [34,35,36,37,38,39,40] in the thermal treatment of liquids from impurities, producing syngas from coal–water compositions, and waste-heat recovery in contact water heaters and superheaters.

## 4. Conclusions

1. Using the experimental results obtained by the optical techniques of PLIF and LIP, we have confirmed the hypotheses previously formulated by the authors of the manuscript (discussed in papers [22,23,24,31]) about a highly unsteady and inhomogeneous temperature field of a rapidly-evaporating water droplet, as well as a gas-vapor mixture around it and behind it (i.e., in its trace) with the incoming high-temperature gas flow.
2. Using PLIF, we established that the temperature field of a droplet becomes quasi-steady only after 10%–15% of its complete evaporation time. The temperature trace of an evaporating droplet (i.e., a gas-vapor cloud) remains inhomogeneous due to a decrease in the droplet dimensions during evaporation, as well as in the convective mixing of cold water vapors and heated combustion products. By analyzing two main mechanisms of decreasing the temperature of the gas-vapor mixture in the trace of a water droplet, we established that the higher the temperature of the incoming gas flow, the more significantly it falls in the trace of an evaporating droplet. The endothermic phase transition plays a major role. Due to the convective heat exchange, it is difficult to considerably decrease the temperature in the droplet trace.
3. A new approach has been proposed to the description of temperature fields in a system with rapidly-evaporating droplets of liquid. It is based on the experimental recording of temperature in PLIF and LIP availability zones with subsequent mathematical modeling of temperature fields of a small region inaccessible for these techniques. When modeling the corresponding problems of heat transfer in the boundary conditions, we used the temperature values determined in the experiments. The research findings are a backbone for the development of modern models of vaporization in the region of high temperatures with due consideration of the decisive influence of the thermal (vapor) insulation layer of an evaporating water droplet. Of particular value are the established consistent patterns of the processes of the buffer vapor layer formation around an evaporating droplet.

## Nomenclature

*C*_v_	water vapor concentration, %;
*h*_tt_	width of temperature trace of water droplet, mm;
*h* ^v^_tt_	visible width of temperature trace of water droplet, mm;
*h*_vt_	width of aerodynamic (velocity) trace of water droplet, mm;
*l*_tt_	length of temperature trace of water droplet, mm;
*l*_vt_	length of aerodynamics (velocity) trace of water droplet, mm;
*r*	radial coordinate, mm;
*R*_d_	initial droplet radius, mm;
*T*	temperature, °C;
*T*_a_	air temperature, °C;
*T*_d_	temperature inside of water droplet, °C;
*T*_s_	temperature of droplet surface, °C;
*T*_St.Dev_	standard deviation of LIP method, °C;
*T*_t_	temperature of vapor-air mixture in the trace of water droplet, °C;
Δ*T*	temperature drop in the droplet trace as compared to incoming air flow temperature (*T*_a_–*T*_t_), °C;
*t*	time, s;
*U*_a_	velocity of air flow, m/s;
*U*_t_	velocity of vapor-air mixture in the trace of water droplet, m/s;
Δ*U*	parameter for air velocity drop in the droplet trace as compared to incoming air flow velocity (*U*_a_–*U*_t_), m/s;
*V*_d_	initial volume of water droplet, μL;
*y*	coordinate, mm;
ν_a_	kinematic viscosity of air, m^2^/s;
Re_a_	Reynolds number for air.
